# On the origin and evolution of biosynthetic pathways: integrating microarray data with structure and organization of the Common Pathway genes

**DOI:** 10.1186/1471-2105-8-S1-S12

**Published:** 2007-03-08

**Authors:** Marco Fondi, Matteo Brilli, Renato Fani

**Affiliations:** 1 Dipartimento di Biologia Animale e Genetica, Università di Firenze, Via Romana 17\19, Firenze, Italy

## Abstract

**Background:**

The lysine, threonine, and methionine biosynthetic pathways share the three initial enzymatic steps, which are referred to as the Common Pathway (CP). In *Escherichia coli *three different aspartokinases (AKI, AKII, AKIII, the products of *thrA*, *metL *and *lysC*, respectively) can perform the first step of the CP. Moreover, two of them (AKI and AKII) are bifunctional, carrying also homoserine dehydrogenasic activity (*hom *product). The second step of the CP is catalyzed by a single aspartate semialdehyde dehydrogenase (ASDH, the product of *asd*). Thus, in the CP of *E. coli *while a single copy of ASDH performs the same reaction for three different metabolic routes, three different AKs perfom a unique step. Why and how such a situation did emerge and maintain? How is it correlated to the different regulatory mechanisms acting on these genes? The aim of this work was to trace the evolutionary pathway leading to the extant scenario in proteobacteria.

**Results:**

The analysis of the structure, organization, phylogeny, and distribution of *ask *and *hom *genes revealed that the presence of multiple copies of these genes and their fusion events are restricted to the γ-subdivision of proteobacteria. This allowed us to depict a model to explain the evolution of *ask *and *hom *according to which the fused genes are the outcome of a cascade of gene duplication and fusion events that can be traced in the ancestor of γ-proteobacteria. *Moreover*, the appearance of fused genes paralleled the assembly of operons of different sizes, suggesting a strong correlation between the structure and organization of these genes. A statistic analysis of microarray data retrieved from experiments carried out on *E. coli *and *Pseudomonas aeruginosa *was also performed.

**Conclusion:**

The integration of data concerning gene structure, organization, phylogeny, distribution, and microarray experiments allowed us to depict a model for the evolution of *ask *and *hom *genes in proteobacteria and to suggest a biological significance for the extant scenario.

## Background

**Figure 1:**
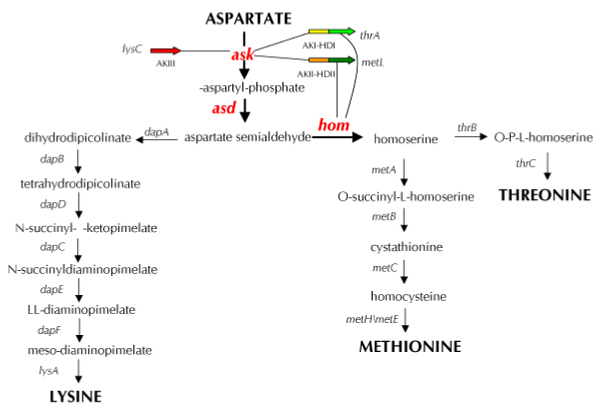
**The aspartate pathway**. Genes marked in red (*ask*, *asd*, and *hom*) constitute the Common Pathway [1].

The metabolic routes leading to the synthesis of lysine\diaminopimelic acid, methionine and threonine\isoleucine are closely interconnected forming a complex system, three steps of which represent the so-called Common Pathway (CP) [1] (Figure 1). The first of them is the phosphorylation of aspartate, carried out by an aspartokinase (AK, the product of the *ask *gene) leading to β-aspartyl-phosphate, which, in turn, is oxidised by an aspartate semialdehyde dehydrogenase (ASDH, the enzyme encoded by *asd*) to aspartate semialdehyde that, finally, may be transformed either into dihydrodipicolinate, the precursor of diaminopimelic acid and lysine, by dihydrodipicolinate synthase (coded for by *dapA*) or homoserine by homoserine dehydrogenase (HD, encoded by *hom*). Homoserine can be then channeled towards threonine and/or methionine biosyntheses. From an evolutionary point of view, the genes coding for these three enzymes are particularly interesting, since at least two different molecular mechanisms, i.e. paralogous gene duplication and gene fusion, appeared to have played a key role in their origin and evolution. In addition to this, in some bacteria each CP step is catalyzed by enzymes coded for by single monofunctional genes, whereas in the enterobacterium *Escherichia coli *it has been shown [2] (Figure 1) that:

i) the first step of the CP can be performed by three different aspartokinases (AKI, AKII and AKIII);

ii) the second step is catalyzed by a monofunctional ASDH encoded by *lysC*; and, lastly,

iii) the third step is carried out by two different homoserine dehydrogenases, referred to as HDI and HDII, which are fused to two of the three AKs: AKI and AKII, respectively. These two bifunctional proteins are coded for by two genes, *thrA *and *metL*, respectively.

The expression of the two *E. coli *bifunctional proteins are differently regulated: threonine and isoleucine regulate the expression of *thrA*, and threonine controls both enzymatic activities by a negative feedback. The transcription of *metL *is repressed by methionine but no feedback inhibition, by methionine itself, has been observed on this enzyme. Finally, the expression of the gene coding for AKIII (*lysC*) and the activity of its product, are regulated in response to lysine concentration [2].

This particular structure pattern has raised the question of how and why it emerged in the course of evolution. On the basis of limited sequence data, Cassan et al. [3] proposed that the present-day bifunctional enzymes may have arisen from a fusion event involving the AK and the HD ancestral coding genes. The duplication of this bifunctional gene may have originated two redundant copies carrying both AK and HD activity. Another gene duplication event may have led to the formation of the three AK copies we observe nowadays. According to this model, the monofunctional AK could have emerged in two different ways: either by a partial gene duplication event involving only the AK activity coding region of the bifunctional genes, or by inactivation, as a result of accumulation of mutations, of the HD coding sequence. Thus, both paralogous gene duplication and gene fusion might have been responsible for shaping the CP. The importance of gene duplication in the course of evolution of genomes and metabolic pathways is well established, (see [4] and references therein): the production of two copies of a DNA sequences leads to an increase of genome size, and it also allows the rapid diversification of enzymatically catalyzed reactions, providing new material for the invention of new enzymatic properties and complex regulatory and developmental patterns. In addition to gene duplication, (see [4] and references therein), one of the major routes of gene evolution is the fusion of independent cistrons leading to bi- or multifunctional proteins [5-9]. Gene fusions provide a mechanism for the physical association of different catalytic domains or of catalytic and regulatory structures [5]. Fusions frequently involve genes coding for proteins functioning in a concerted manner, such as enzyme catalyzing sequential steps within a metabolic pathway [10]. Fusion of such catalytic centres likely promotes the channelling of intermediates that may be unstable and/or in low concentration [5]; this, in turn, requires that enzymes catalysing sequential reactions are colocalized within cell [11] and may (transiently) interact to form complexes that are termed metabolons [12]. The high fitness of gene fusions can also rely on the tight regulation of the expression of the fused domains. This might be the case of *metL *and *thrA*.

Thus, the CP might represent a very interesting model study to shed some light on the mechanisms driving the assembly of metabolic pathways and the refinement of regulatory networks. Nonetheless, in spite of the availability of several completely sequenced genomes and microarray data, neither a detailed analysis of the structure and organization of CP genes has been carried out nor any correlation of these data with expression (microarray) ones has been established until now. The aim of this work was to try to reconstruct the possible evolutionary and timing pathway(s) leading to the extant *ask *and *hom *genes, to analyse their phylogenetic distribution, to shed some light on the molecular mechanisms responsible for the assembly of the CP genes in bacteria and on the role that gene duplication(s), fusion(s) and clustering might have had in this context. To this purpose, the structure, organization and phylogenetic distribution of all the available proteobacterial *ask*, *hom*, and *asd *genes were analysed. Data obtained were integrated with expression data deriving from microarray analyses. We focused our attention on Proteobacteria for the following reasons: i) previous works [6,7,9] have shown that gene rearrangement events, such as gene duplication, fusion, and/or clustering have strongly influenced their evolution, ii) this phylogenetic branch includes the γ-subdivision, that is thought to be one of the most recent branching point among Bacteria and iii) they represent a good case-study since comprise organisms living in very different habitats (going from the deep-sea hydrothermal environments of the ε-subdivision to the roots of plants in the case of some α-proteobacteria), and with very different lifestyles, including endosymbionts and parasites.

## Results and discussion

### Structure and phylogenetic distribution of the genes coding for AK, ASDH and HD in Proteobacteria

**Figure 2:**
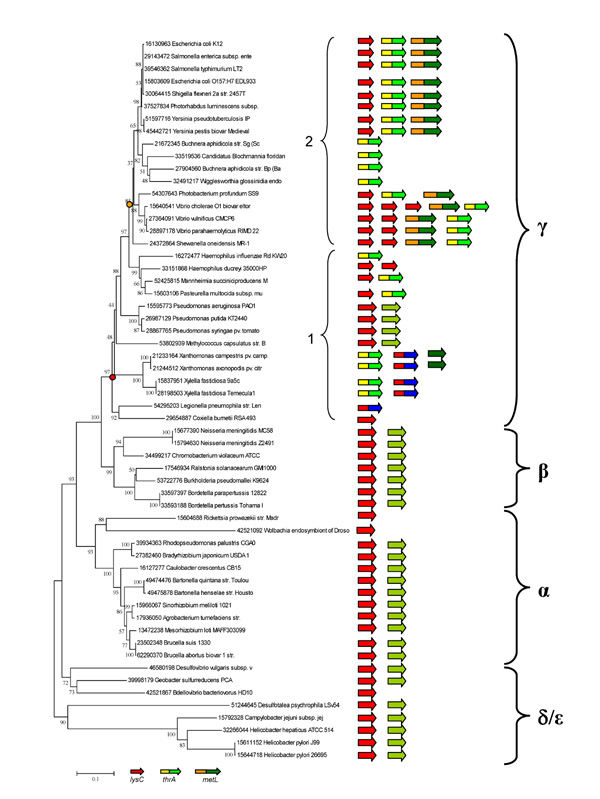
**The structure of *ask *and *hom *genes**. Phylogenetic tree constructed using the RpoD sequences (Neighbor Joining, 2250 Boostrap Replicates, Complete Deletion, Poisson Correction) of the 58 proteobacteria together with the number and the structure of all the retrieved *ask *and *hom *genes.

The aminoacid sequences of the *E. coli *AK, ASDH, and HD sequences were used as a query to probe the protein database of completely sequenced proteobacterial genomes with the BLASTP option of BLAST program [13], in order to retrieve the most similar sequences. To this purpose 58 proteobacterial genomes were selected and, in most cases, only one strain for each species was taken into account. Data obtained are schematically reported in Figure 2, where a phylogenetic tree constructed using the RpoD sequences of the 58 proteobacteria is shown together with the number and the structure of all the retrieved AK, and HD coding genes. The *asd *genes were not included in Figure 2, since just one copy of this gene was retrieved from the 58 proteobacteria. The analysis of data reported in Figure 2 revealed that:

a) in all the *α*-, *β*- and *δ\ε*-proteobacterial genomes a single, monofunctional, stand-alone, copy of the gene coding for AK or HD was detected; moreover, neither duplicated copies nor fusion events involving these genes were detected.

b) multiple as well as fused copies of AK and HD were found only in γ-proteobacteria, where the scenario is (apparently) more complex and intriguing. Indeed, a variable structure and copy-number of genes coding for AK (1 to 5) and HD (1 to 2) can be observed. Moreover, there is an apparent increasing complexity concerning these genes that is parallel to the evolutionay branching of γ-proteobacteria, with enterobacteria and vibrionaceae showing the highest number of redundant and fused copies of AK and HD. This phylogenetic distribution strongly suggests that the duplication of AK coding genes and the fusion to HD apparently can be traced within γ-proteobacteria or soon after the divergence of the γ-proteobacterial ancestor from *α*-, *β*- and *δ\ε*-proteobacteria.

### A model for the evolution of the AK and HD coding genes

**Figure 3:**
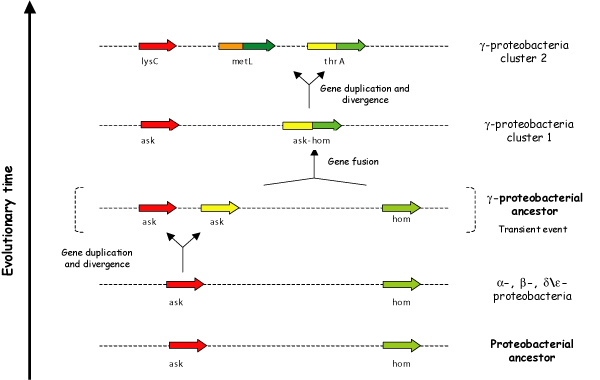
**The evolutionary model**. Evolutionary model proposed to explain the evolution of *ask *and *hom *genes in proteobacteria.

On the basis of the phylogenetic distribution of stand-alone and bifunctional genes of the CP we propose a possible, plausible evolutionary and timing model explaining the extant scenario. The model, which is schematically reported in Figure 3, predicts that the proteobacterial ancestor possessed a single copy of *hom*, *ask *and *asd *genes. During evolution, this organization was maintained in proteobacteria belonging to the *α*-, *β*- and *δ\ε*-subdivisions. One of the cross-roads for the evolution of these genes is represented by the branching point between β- and γ-proteobacteria. It appears quite possible that, in the ancestor of γ-proteobacteria, a first duplication of the *ask *gene may have taken place, generating two redundant copies that underwent an evolutionary divergence. The finding that no bacterium (with the exception of *Vibrio *strains, see below) shows two copies of monofunctional *ask *genes, strongly suggests that this duplication event and its further fusion to *hom *might have occurred in a relatively short evolutionary time, giving raise to an ancestral bifunctional gene, which might have retained the function of the extant *metL *and *thrA*. This sort of "gene duplication-gene fusion coupling" is quite similar to that described recently for the evolution of γ-proteobacterial *hisN *and *hisB *histidine biosynthetic genes [6,7,9]. Finally, a paralogous duplication event of this bifunctional ancestor gene followed by evolutionary divergence (which very likely concerned with the regulatory mechanism, rather than the catalytic activity) led to the extant *metL *and *thrA *genes. On the basis of the phylogenetic distribution of the bifunctional genes (Figure 3), this "final" step might have occurred just before the separation between the "clusters" 1 and 2 of the γ-proteobacterial subdivision.

The biological significance of this cascade of duplication and fusion events might rely on the "patchwork" hypothesis on the origin and evolution of metabolic pathways [14]. According to this idea, metabolic pathways may have been assembled through the recruitment of primitive enzymes that could react with a wide range of chemically related substrates. Such relatively slow, unspecific enzymes may have been enabled primitive cells containing small genomes to overcome their limited coding capabilities [4]. Paralogous gene duplication event(s) followed by evolutionary divergence might have permitted the appearance of enzymes with an increase and narrow specificity and/or the diversification of function. In this way, an ancestral enzyme belonging to a given metabolic route, is "recruited" to serve a single or other (novel) pathways. Besides, it may permit the *evolution and refinement of regulatory mechanisms* coincident with the development of new pathways and/or the refinement of pre-existing ones.

In our opinion, the evolutionary model proposed here to explain the origin and evolution the extant *metL *and *thrA *genes is in full agreement with the Jensen hypothesis and the cascade of gene duplications and fusions involving *ask *and *hom *genes might actually represent a mechanism for the refinement of the feedback regulation mechanisms controlling the activity of the enzymes they code for.

### Phylogenetic analysis

**Figure 4:**
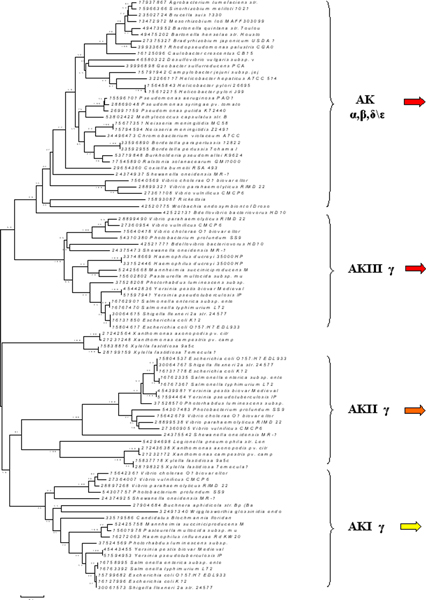
**Phylogenetic tree of AK sequences**. Phylogenetic trees (Neighbor Joining, 2250 Boostrap Replicates, Complete Deletion, Poisson Correction) constructed with all the retrieved sequences of AK.

**Figure 5:**
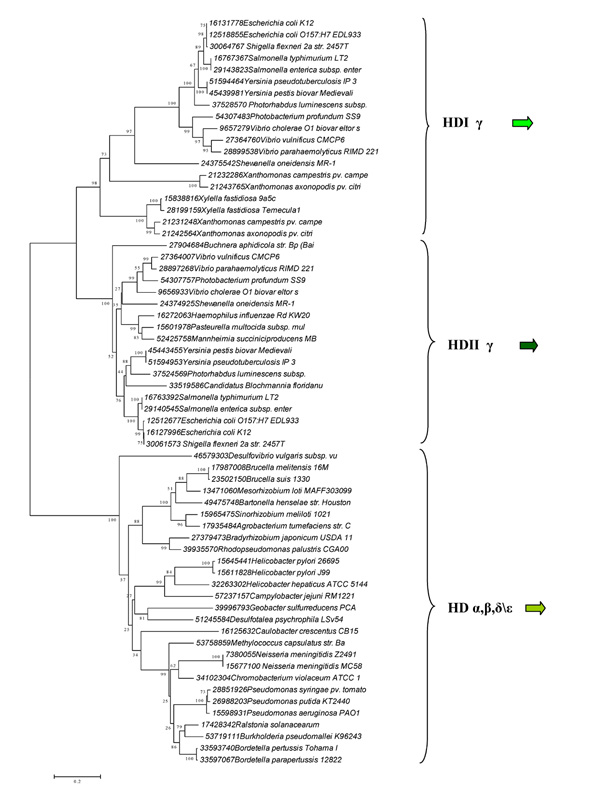
**Phylogenetic tree of HD sequences**. Phylogenetic trees (Neighbor Joining, 2250 Boostrap Replicates, Complete Deletion, Poisson Correction) constructed with all the retrieved sequences of HD.

If the evolutionary model proposed here is correct, one should expect that the fused copies of AK (AKI and AKII) and HD (HDI and HDII) share a degree of sequence similarity higher than that exhibited with AKIII and HD, respectively, and cluster together in a phylogenetic tree. In order to check this hypothesis, the AK and HD aminoacid sequences were aligned using the program ClustalW [15] and the multialignments obtained used to draw the phylogenetic trees shown in Figure 4 and 5. The analysis of the AK tree (Figure 4) showed that all the α-, β- and δ\ε-proteobacterial sequences form a unique cluster separated from γ-proteobacterial ones. Besides, the γ-proteobacterial AKI, AKII, and AKIII sequences form three different and separated clusters with AKIII representing the root of the others. A similar situation can be observed in the HD tree (Figure 5): α-, β- and δ\ε-proteobacterial HD sequences form a distinct unique cluster, while HDI and HDII form two close clusters.

The topology of the phylogenetic trees obtained fits well with the evolutionary model proposed and indicates that horizotal gene transfer of these genes rarely occurred and did not strongly influenced the evolution of AK and HD domanis. However, even though the evolutionary model reported in Figure 3 is in agreement with gene structure and phylogenetic analyses, the following exceptions have to be explained:

1) The absence of *lysC *and *metL *in a group of enterobacteria (*Buchnera aphidicola *strains, *Candidatus Blochmannia floridanus*, *Wigglesworthia glossinidia*) and in *Haemophilus influenzae*, the absence of bifunctional genes in *H. ducrey*, and the lack of *hom *in *Coxiella burnetii*, *Ricketsia prowazekii*, *Wolbachia endosymbiont of Drosophila melanogaster *and *Bdellovibrio bacteriovorus*. This is very likely due to the absence of the corrensponding metabolic route(s), which, in turn, is correlated to the parasitic lifestyle of these proteobacteria. Such a lifestyle may allow the bacteria to acquire essential compounds directly from the metabolic activities of their host and the adaptation to this environmental condition might have caused the loss of entire metabolic routes or part thereof.

2) The increase of the AK copies in *Vibrio *strains in respect to other γ-proteobacteria is probably related to the high genomic rearrangement rate typical of these species.

3) The absence of bifunctional *ask-hom *genes in *Pseudomonas *and *Methylococcus capsulatus *that, in spite of their taxonomical position within γ-proteobacteria, exhibit the same structural and organization pattern of bacteria belonging to the *α*-, *β*- and *δ\ε*-subdivisions. This is not an isolated example; in fact, the same situation has been recorded for other biosynthetic pathways, such as histidine biosynthesis [6,7]. The reason(s) of such structure and organization is still unclear.

4) The fusion of *ask *to *lysA *in *Xanthomonadaceae*, which represents an exception to this general model. In these bacteria the paralogous duplication of *ask *gene originated two copies, one of which fused to *hom*, whereas the other one underwent another fusion event with *lysA*, a gene coding coding for DAPDC activity). The biological significance of the last fusion might rely in the spatial colocalization of the products of the two modules and a faster feedback inhibition of the first enzyme (AK) by the end product of the pathway (lysine), whose last biosynthetic step is catalyzed by the enzyme coded for by *lysA*.

### Analysis of gene organization

**Figure 6:**
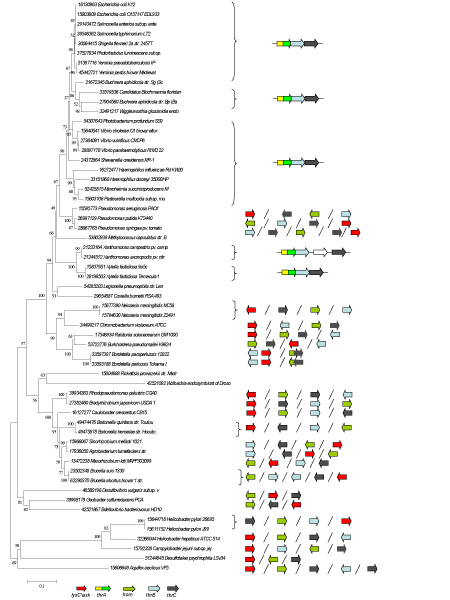
**Gene organization of threonine genes**. Structure and organization of threonine biosynthetic genes of the 58 proteobacteria correlated with their phylogenetic position as established by RpoD analysis.

**Figure 7:**
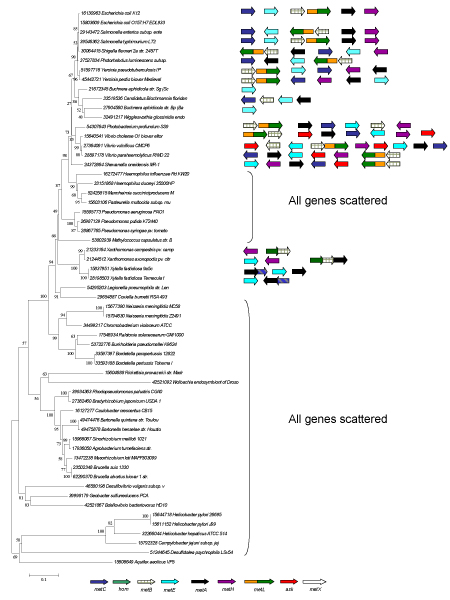
**Gene organization of methionine genes**. Structure and organization of methionine biosynthetic genes of the 58 proteobacteria correlated with their phylogenetic position as established by RpoD analysis.

If the model proposed and its biological significance is correct, i.e. that the duplication and fusion events, and the successive evolutionary divergence allowed the three copies of AKs and the two of HDs to narrow their specificity and to become increasingly more sensitive to specific regulatory signals, then it is plausible to assume that the ancestral copy of AK (AKIII) might serve different metabolic pathways and hence might have been under the control of multiple different regulatory signals (i.e. the availability of DAP, lysine, threonine, methionine etc). On the other hand, the expression of the bifunctional genes, *thrA *and *metL*, once they were channelled towards the biosynthesis of threonine and methionine, should have become increasingly more dependent on more specific signals (for example the concentration of the final product of that route). In general, it is plausible that once a "new" gene introgresses and becomes part of a pre-existing metabolic pathway, it will become co-regulated with the other genes belonging to the same metabolic pathway. In some cases, co-regulation of genes of the same biosynthetic route is achieved by organizing genes in operon structures, even though co-regulation may also be obtained by regulon construction. This is particularly true for fused genes; as reported in previous works, based on the analysis of the histidine biosynthetic pathway in γ-proteobacteria, the appearance of fused genes (specific for a single pathway) is often parallel to their presence within operons [6,7,9]. This raises the question whether the structure and distribution of duplicated and fused copies of *ask *and *hom *genes might somehow be correlated to their organization in the proteobacterial genome. Therefore, we analysed the organization of all the genes of the *lys*, *met *and *thr *biosynthesis in all the 58 proteobacteria. Data obtained revealed that:

1. Genes involved in the DAP\lysine biosynthesis are scattered throughout the chromosome(s) of all the 58 proteobacteria taken into account (data not shown).

2. In addition to *ask*, *asd *and *hom *genes, the other two genes involved in threonine biosynthesis (*thrB *and *thrC*) are scattered on the chromosome of bacteria belonging to α-, β- and δ\ε subdivisions (except *Bordetella *strains that own a *hom-thrC *operon) (Figure 6). The γ-proteobacterial scenario is completely different; according to the hypothesis mentioned above, in all of organisms possessing a bifunctional *thrA *gene, it is endowed within a three-cystronic operon, in the same relative gene order (*thrABC*), also suggesting that its construction should have been occurred once during evolution.

3. The organization of methionine biosynthetic genes in proteobacteria partly reflects that exhibited by *lys *or *thr *genes. In fact, in the α-, β- and δ\ε branches all the *met *biosynthetic genes are scattered on the chromosome(s) (Figure 7). This organization is also shared by γ-proteobacteria; the only exception is represented by the bifunctional *metL*, which is clustered with *metB *to form a bicistronic *metLB *operon.

Thus, no bifunctional gene of the CP is located outside operons. Data obtained strongly suggest that the production of genes coding for enzymes specific of a single metabolic pathway coincides with their presence within a polycistronic transcriptional unit that includes all (or at least some of) the other genes of that route. Concerning the timing of the operons construction, the comparative analysis of Figure 2, 5, and 6 revealed that the "gene duplication-gene fusion coupling" occurring in γ-proteobacteria appears to be coincident with gene clustering and the formation of operons of different length.

### Analysis of microarray experiments data

In order to elucidate the correlation existing between the structure and organization of *lys*, *met*, and *thr *genes and their expression within the cell, we analyzed the microarray data from *E. coli *and *P. aeruginosa*, which show two different arrays of structure and organization of CP genes. Microarray data were downloaded as supplemental material to published papers (see Additional File 1: Additional References for the Expression compendium); only normalized and filtered data were used. Values were transformed into base 2 logarithm of the ratio of the wild type (untreated) / mutant (treated) expression levels, if not yet in that form.

For each of the three metabolic pathways we carried out a pairwise comparison of the expression pattern of each gene, by calculating the Pearson's correlation coefficient.

**Figure 8:**
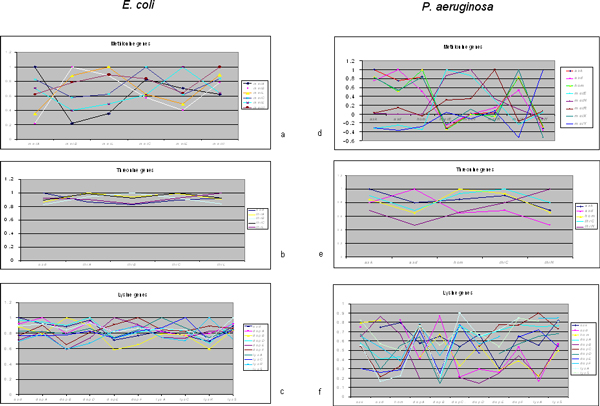
**Microarray data analysis**. Comparison between the expression pattern of each *met*, *lys*, *thr *gene of *E. coli *(a, b, c) and *P. aeruginosa *(d, e, f).

Data obtained are reported in Figure 8, whose analysis revealed:

1. A low co-regulation of the methionine biosynthetic genes (Figure 8a). Most of these genes are scarcely co-expressed, and they appeared to be expressed independently from each other. The fact that both *metL *and *metB *show very high correlation coefficient value in respect to the other *met *genes is in agreement with their operonic organization.

2. The three *E. coli thrABC *genes (Figure 8b) are highly co-expressed, with correlation coefficient > 0.84. This is in agreement with their organization in a compact operon.

3. The trend of the lysine pathway genes in the γ-proteobacterium *E. coli *(Figure 8c) is quite surprising; although the *lys *genes are scattered throughout the *E. coli *chromosome, they show a high degree of co-expression with correlation coefficient values often > 0.8. It is not clear how these genes can be highly co-expressed in the absence of an operonic organization. However, it is known [16] that lysine biosynthetic genes are regulated by the so-called LYS *element *(lysine-specific RNA element) located in their regulatory regions and able to repress or to allow their trascription in response to lysine concentration. The high coexpression pattern of lysine bosynthetic genes might be due to this mechanism.

The same analysis was carried out on lysine, methionine and threonine biosynthetic genes of *Pseudomonas aeruginosa*, whose structure and organization pattern is the same of the α-, β-, and δ\ε subdivision of proteobacteria. Data obtained (reported in Figure 8) showed that, overall, there is a low degree of co-expression between genes belonging to the same pathway; this is particularly pronounced for methionine, where in some cases, the correlation coefficient assumes negative values (Figure 8e), and lysine genes, whereas the *thr *biosynthetic genes were more correlated between them. The low degree of co-expression of *P. aeruginosa *genes is in agreement with their scattering on the bacterial genome.

## Conclusion

In this work a likely model for the evolution of the genes involved in the common pathway (CP) is depicted, which is based on the comparative analysis of data concerning the structure, phylogenetic distribution, organization, phylogeny and expression of *ask *and *hom *genes in proteobacteria. The analysis of the structure of the CP genes gave a strong support to the hypothesis that at least two different molecular mechanisms played an important role in shaping the pathway, that is paralogous gene duplication(s) and gene fusion [17,4]. The analysis of *thr*, *met *and *lys *gene organization in different proteobacteria revealed that several gene arrays exist within this phylogenetic lineage, with genes completely scattered throughout the genome, partially scattered/clustered, or strictly compacted. Even though different scenarios can be depicted for this different organization, i.e. the presence of scattered or clustered genes in the ancestor of proteobacteria, data reported in this work supported the first hypothesis. According to the model proposed, the ancestor of proteobacteria possessed monofunctional *hom*, *ask*, and *asd *genes scattered throughout the genome. The extant multiple and fused copies of *ask *and *hom *genes are the outcome of a cascade of paralogous gene duplication and fusion events, which led to the appearance of bifunctional enzymes catalyzing the same metabolic steps, but "sensing" different regulatory signals.

The evolutionary history of the CP genes gives another important support to the Jensen's hypothesis on the origin and evolution of metabolic pathways [14], strengthening the idea that gene duplication, gene fusion and recruitment of genes encoding enzyme with a broad range of substrate specificity played a crucial role in the assembly of biosynthetic pathways and in the appearance of new and/or more sophisticated regulatory networks [4,9]. Indeed, the biological significance of the presence of multiple copies of *ask *and *hom *genes might rely on the refinement of regulatory mechanisms allowing each *ask *copy to be regulated by specific signals, such as the availability of the end-product of the pathway.

The question of why the duplicated copies of *ask *fused to *hom *is rather intriguing. It is evident from their phylogenetic distribution that, once occurred, the fusion has been fixed; thus, it should have been evolutionary advantageous. Even though it cannot be *a priori *excluded, we do not favour the possibility that this fusion might permit the substrate tunnelling. It is possible that this gene fusion (and gene organization) resulted from both regulatory and metabolic constraints, for instance it might permit the spatial colocalization of their products and so a faster feedback inhibition of the first enzyme of the pathway, coded for by *ask*, by the product of *hom*.

The existence of the *thrA and metL *gene fusions in the genome of γ-proteobacteria is not an isolated example; additional gene fusions occurred in these genomes, such as those involving some histidine biosynthetic genes. It is worth of note that most of bifunctional proteins recognized to date are involved in metabolic pathways of the γ-subdivision of proteobacteria [18]. Even though there is no apparent reason to think that these organisms are more prone to gene fusions than any others, it is interesting that these gene fusions appeared to be parallel to the increasing compactness of some operons [9] or to their construction, as in the case of the *thrABC *and *metLB *ones.

Actually, the analysis of the organization of these genes revealed that all the *metL *and *thrA *genes are embedded within (compact) operons, whereas their monofunctional counterparts as well as the second CP gene, *asd*, are located outside gene clusters. This is not so surprising if we agree on the existence of unspecific enzymes that might serve different metabolic pathways. Indeed, it is plausible that the expression of a gene, whose product catalyses a chemical reaction leading to a product involved in different metabolic pathways should be constitutively expressed or controlled by multiple mechanisms rather than being controlled by mechanisms specific for a single route.

This is also in agreement with expression data retrieved from the available microarray data; in fact, the greater the scattering of genes belonging to the same pathway, the lower the degree of correlation between them.

If our model is correct, the building up of *thrABC *and *metLB *operons represents a recent invention of evolution (dated in the γ proteobacterial ancestor) and is apparently co-incident with the appearance of bifunctional *ask*-*hom *genes. The origin and evolution of operons is still under debate, and at least six different classes of models have been proposed to explain the existence of operons (see [9] and references therein); although different forces might have driven the assembly of operons, in our opinion the major ones were those enabling the *fused *genes to be coregulated finely and the protein coded for synthesized in the correct stoichiometric ratio.

## Material and methods

### Sequence retrieval

Amino acid sequences were retrieved from GenBank database. BLAST [13] probing of database was performed with the BLASTP option of this program using default parameters. Only those sequences retrieved at an E-value below the 0.05 threshold were taken into account.

### Sequence alignment

The ClustalW [15] program in the BioEdit package was used to perform pairwise and multiple amino acid sequences alignments.

### Phylogenetic trees construction

Phylogenetic trees were obtained with Mega 3 [19] software using the Neighbor-Joining (NJ) and the Minimum Evolution (ME) methods.

## List of abbreviations

AKI, AKII, AKIII, Aspartokinase I, II, III; *askI *and *askII *can also be named as *thrA *and *metL*; ASHD, Aspartate semialdehyde dehydrogenase; DAPDC, meso-diaminopimelate decarboxylase; HD, homoserine dehydrogenase.

## Authors' contributions

All authors equally contributed to the preparation of the final version of the manuscript; MF performed the analyses during its MS degree work under the supervision of Prof. RF.

## Supplementary Material

Additional File 1 Additional References for the Expression compendium. List of the references used to retrieve microarray experiments data. Click here for file
