# Supplementary material for: On the origin and evolution of biosynthetic pathways: integrating microarray data with structure and organization of the Common Pathway genes
Source: BMC Bioinformatics. 2007 Mar 8;8(Suppl 1):S12. doi: 10.1186/1471-2105-8-S1-S12 (PMC1885841; doi:10.1186/1471-2105-8-S1-S12)
Supplement: Additional File 1 — Additional References for the Expression compendium. List of the references used to retrieve microarray experiments data. [file 1471-2105-8-S1-S12-S1.pdf]

Additional References for the Expression compendium

Chang W, Small DA, Toghrol F, Bentley WE: **Microarray analysis of *Pseudomonas aeruginosa* reveals induction of pyocin genes in response to hydrogen peroxide.** *BMC Geno*mics 2005, **6**:115.

Chang W, Small DA, Toghrol F, Bentley WE: **Microarray Analysis of Toxicogenomic Effects of Peracetic Acid on *Pseudomonas aeruginosa***. *Environ. Sci. Technol.* 2005, **39**:5893-5899.

Courcelle J, Khodursky A, Peter B, Brown PO, Hanawalt PC: **Comparative gene expression profiles following UV exposure in wild-type and SOS-deficient *Escherichia coli***. *Genetics* 2001, **158**:41-64.

Covert MW, Knight EM, Reed JL, Herrgard MJ, Palsson BO: **Integrating high-throughput and computational data elucidates bacterial networks.** *Nature* 2004, **429**(6987):92-96.

Hung SP, Baldi P, Hatfield GW: **Global gene expression profiling in *Escherichia coli* K12. The effects of leucine-responsive regulatory protein**. *J Biol Chem.* 2002, **277**(43):40309-40323.

Kabir MS, Yamashita D, Koyama S, Oshima T, Kurokawa K, Maeda M, Tsunedomi R, Murata M, Wada C, Mori H, Yamada M: **Cell lysis directed by sigmaE in early stationary phase and effect of induction of the *rpoE* gene on global gene expression in Escherichia coli**. *Microbiology* 2005, **151**(Pt 8):2721-2735.

Khodursky AB, Peter BJ, Cozzarelli NR, Botstein D, Brown PO, Yanofsky C: **DNA microarray analysis of gene expression in response to physiological and genetic changes that affect tryptophan metabolism in *Escherichia coli*.** *Proc. Natl. Acad. Sci. U. S. A.* 2000, **97**(22):12170-12175.

Kuchma SL, Connolly JP, O'Toole GA: **A Three-Component Regulatory System Regulates Biofilm Maturation and Type III Secretion in *Pseudomonas aeruginosa.*** *J Bacteriol* 2005,**187**:1441–1454.

Lee K, Bernstein JA, Cohen SN: **RNase G complementation of rne null mutation identifies functional interrelationships with RNase E in *Escherichia coli*.** *Mol Microbiol.* 2002, **43**(6):1445-1456. *Erratum in: Mol Microbiol* 2002,**46**(1):295.

Lee LJ, Barrett JA, Poole RK: **Genome-wide transcriptional response of chemostat-cultured Escherichia coli to zinc**. *J Bacteriol.* 2005, **187**(3):1124-1134.

Liu M, Durfee T, Cabrera JE, Zhao K, Jin DJ, Blattner FR: **Global transcriptional programs reveal a carbon source foraging strategy by Escherichia coli**. *J Biol Chem.* 2005, **280**:15921-15927.

Lobner-Olesen A, Marinus MG, Hansen FG: **Role of SeqA and Dam in *Escherichia coli* gene expression: a global/microarray analysis**. *Proc Natl Acad Sci U. S. A.* 2003, **100**(8):4672-4677.

Matthew C, Wolfgang MC, Jyot J, Goodman AL, Rampha R, Lory S: ***Pseudomonas aeruginosa* regulates flagellin expression as part of a global response to airway fluid from cystic fibrosis patients.** *Proc. Natl. Acad. Sci. U. S. A.* 2004, **101**:6664–6668.

Nakahigashi K, Kubo N, Narita S, Shimaoka T, Goto S, Oshima T, Mori H, Maeda M, Wada C, Inokuchi H: **HemK, a class of protein methyl transferase with similarity to DNA methyltransferases, methylates polypeptide chain release factors, and hemK knockout induces defects in translational termination**. *Proc. Natl. Acad. Sci. U. S. A.* 2002, **99**(3):1473-1478.

Oh MK, Rohlin L, Kao KC, Liao JC: **Global expression profiling of acetate-grown *Escherichia coli*.** *J. Biol. Chem.* 2002, **277**(15):13175-13183.

Oshima T, Aiba H, Masuda Y, Kanaya S, Sugiura M, Wanner BL, Mori H, Mizuno T: **Transcriptome analysis of all two-component regulatory system mutants of *Escherichia coli* K-12**. *Mol. Microbiol.* 2002, **46**(1):281-291.

Salmon K, Hung SP, Mekjian K, Baldi P, Hatfield GW, Gunsalus RP: **Global gene expression profiling in *Escherichia coli* K12. The effects of oxygen availability and FNR.** *J. Biol. Chem.* 2003, **278**(32):29837-29855.

Wolfgang CM, Lee VT, Gilmore ME, Lory S: **Coordinate Regulation of Bacterial Virulence Genes by a Novel Adenylate Cyclase-Dependent Signaling Pathway**. *Developmental Cell* 2003, **4**:253–263.
